# Supplementary material for: Geometric morphometric analysis of morphologic disparity, intraspecific variation and ontogenetic allometry of beyrichitine ammonoids
Source: PLoS One. 2022 Feb 10;17(2):e0263524. doi: 10.1371/journal.pone.0263524 (PMC8830730; doi:10.1371/journal.pone.0263524)
Supplement: Attachment — Submitted filename: PONE-D-21-30107_reviewer_KDB.pdf [file pone.0263524.s006.pdf]

PLOS ONE

Geometric Morphometric analysis of morphologic disparity, intraspecific variation and ontogenetic allometry of beyrichitine ammonoids

--Manuscript Draft--

**Manuscript Number:**	PONE-D-21-30107
**Article Type:**	Research Article
**Full Title:**	Geometric Morphometric analysis of morphologic disparity, intraspecific variation and ontogenetic allometry of beyrichitine ammonoids
**Short Title:**	Morphologic disparity, intraspecific variation and ontogenetic allometry of ammonoids
**Corresponding Author:**	Eva Alexandra Bischof, PhD<br>Universität Bremen: Universitat Bremen<br>Bremen, Bremen GERMANY
**Keywords:**	Geometric morphometrics; heterochrony; allometry; ontogeny; morphologic disparity; ontogenetic trajectory space; morphospace; landmarks; Beyrichitinae; Anisian; Nevada
**Abstract:**	Beyrichitine ammonoids of NV Nevada reveal a high taxonomic diversity of Anisian (Middle Triassic). This diversity is, however, in contrast to their relatively low morphologic disparity. Depending on the exact definition, morphologic disparity of a data set is a direct consequence of the sum of all ontogenetic changes. In the past, however, the interplay of both morphological processes has only rarely been addressed.<br>Using geometric morphometric methods, this study aims at a quantification of allometric processes and the morphologic disparity of beyrichitine ammonoids. The multivariate statistical analysis revealed that morphologic disparity, intraspecific variation respectively, within and between the studied species seems to be the result of deviations in the ontogenetic allometric growth pattern (i.e. heterochrony). During deposition of the studied stratigraphic sequence, a general progressive pedomorphism (juvenilization) was observed. The intraspecific variability pattern coincides with the total morphologic disparity of the analyzed species, which suggests that intraspecific variability facilitated morphologic disparity. The comparison of ontogenetic allometric patterns and changes in intraspecific variation and morphologic disparity are likely to refine our understanding of the intrinsic factors influencing the speciation of this group.
**Order of Authors:**	Eva Alexandra Bischof
	Nils Schlüter, PhD
	Jens Lehmann, Prof. Dr.
**Additional Information:**	
**Question**	**Response**
**Financial Disclosure**<br><br>Enter a financial disclosure statement that describes the sources of funding for the work included in this submission. Review the submission guidelines for detailed requirements. View published research articles from *PLOS ONE* for specific examples.<br><br>This statement is required for submission and **will appear in the published article** if the submission is accepted. Please make sure it is accurate.	This research received support from the German Science Foundation (DFG), project "Nevammonoidea"' (LE 1241/3-1). The funders had no role in study design, data collection and analysis, decision to publish, or preparation of the manuscript.

Powered by Editorial Manager® and ProduXion Manager® from Aries Systems Corporation

**Unfunded studies**<br>Enter: *The author(s) received no specific funding for this work.*<br><br>**Funded studies**<br>Enter a statement with the following details:<br>• Initials of the authors who received each award<br>• Grant numbers awarded to each author<br>• The full name of each funder<br>• URL of each funder website<br>• Did the sponsors or funders play any role in the study design, data collection and analysis, decision to publish, or preparation of the manuscript?<br>• **NO** - Include this sentence at the end of your statement: *The funders had no role in study design, data collection and analysis, decision to publish, or preparation of the manuscript.*<br>• **YES** - Specify the role(s) played.<br><br>* typeset	
**Competing Interests**<br><br>Use the instructions below to enter a competing interest statement for this submission. On behalf of all authors, disclose any competing interests that could be perceived to bias this work—acknowledging all financial support and any other relevant financial or non-financial competing interests.<br><br>This statement is required for submission and **will appear in the published article** if the submission is accepted. Please make sure it is accurate and that any funding sources listed in your Funding Information later in the submission form are also declared in your Financial Disclosure statement.<br><br>View published research articles from *PLOS ONE* for specific examples.	There are no competing interests.

Powered by Editorial Manager® and ProduXion Manager® from Aries Systems Corporation

**NO authors have competing interests**<br><br>Enter: *The authors have declared that no competing interests exist.*<br><br>**Authors with competing interests**<br><br>Enter competing interest details beginning with this statement:<br><br>*I have read the journal's policy and the authors of this manuscript have the following competing interests: [insert competing interests here]*<br><br>* typeset	
**Ethics Statement**<br><br>Enter an ethics statement for this submission. This statement is required if the study involved:<br><br>• Human participants<br>• Human specimens or tissue<br>• Vertebrate animals or cephalopods<br>• Vertebrate embryos or tissues<br>• Field research<br><br>Write "N/A" if the submission does not require an ethics statement.<br><br>General guidance is provided below. Consult the submission guidelines for detailed instructions. **Make sure that all information entered here is included in the Methods section of the manuscript.**	N/A

Powered by Editorial Manager® and ProduXion Manager® from Aries Systems Corporation

**Format for specific study types**<br><br>**Human Subject Research (involving human participants and/or tissue)**<br>• Give the name of the institutional review board or ethics committee that approved the study<br>• Include the approval number and/or a statement indicating approval of this research<br>• Indicate the form of consent obtained (written/oral) or the reason that consent was not obtained (e.g. the data were analyzed anonymously)<br><br>**Animal Research (involving vertebrate animals, embryos or tissues)**<br>• Provide the name of the Institutional Animal Care and Use Committee (IACUC) or other relevant ethics board that reviewed the study protocol, and indicate whether they approved this research or granted a formal waiver of ethical approval<br>• Include an approval number if one was obtained<br>• If the study involved *non-human primates*, add *additional details* about animal welfare and steps taken to ameliorate suffering<br>• If anesthesia, euthanasia, or any kind of animal sacrifice is part of the study, include briefly which substances and/or methods were applied<br><br>**Field Research**<br><br>Include the following details if this study involves the collection of plant, animal, or other materials from a natural setting:<br>• Field permit number<br>• Name of the institution or relevant body that granted permission	
**Data Availability**<br><br>Authors are required to make all data underlying the findings described fully available, without restriction, and from the time of publication. PLOS allows rare exceptions to address legal and ethical concerns. See the PLOS Data Policy and FAQ for detailed information.	Yes - all data are fully available without restriction

A Data Availability Statement describing where the data can be found is required at submission. Your answers to this question constitute the Data Availability Statement and **will be published in the article**, if accepted.<br><br>**Important:** Stating 'data available on request from the author' is not sufficient. If your data are only available upon request, select 'No' for the first question and explain your exceptional situation in the text box.<br><br>Do the authors confirm that all data underlying the findings described in their manuscript are fully available without restriction?	
**Describe where the data may be found in full sentences. If you are copying our sample text, replace any instances of XXX with the appropriate details.**<br><br>• If the data are **held or will be held in a public repository**, include URLs, accession numbers or DOIs. If this information will only be available after acceptance, indicate this by ticking the box below. For example: *All XXX files are available from the XXX database (accession number(s) XXX, XXX.*).<br>• If the data are all contained **within the manuscript and/or Supporting Information files**, enter the following: *All relevant data are within the manuscript and its Supporting Information files.*<br>• If neither of these applies but you are able to provide **details of access elsewhere**, with or without limitations, please do so. For example:<br><br>*Data cannot be shared publicly because of [XXX]. Data are available from the XXX Institutional Data Access / Ethics Committee (contact via XXX) for researchers who meet the criteria for access to confidential data.*<br><br>*The data underlying the results presented in the study are available from (include the name of the third party*	The data is supplied in the supportin Information (S2)

and contact information or URL).<br>• This text is appropriate if the data are owned by a third party and authors do not have permission to share the data.<br><br>* typeset	
Additional data availability information:	Tick here if the URLs/accession numbers/DOIs will be available only after acceptance of the manuscript for publication so that we can ensure their inclusion before publication.

Powered by Editorial Manager® and ProduXion Manager® from Aries Systems Corporation

Manuscript
Click here to access/download;Manuscript;AA_Manuscript_v6-2.docx

Full title: Geometric Morphometric analysis of morphologic disparity, intraspecific variation and ontogenetic allometry of beyrichitine ammonoids

Short title: Morphologic disparity, intraspecific variation and ontogenetic allometry of ammonoids

Eva A. Bischof[1], Nils Schlüter[2], Jens Lehmann[1]

[1] Geowissenschaftliche Sammlung, Fachbereich Geowissenschaften, Universität Bremen, Leobener Strasse 8, 28357 Bremen, Germany

[2] Naturhistorisches Forschungsinstitut, Museum für Naturkunde, Humboldt-Universität zu Berlin, Invalidenstraße 43, 10115 Berlin, Germany

Corresponding author:
Eva Bischof[1]
Email address: eva.bischof@hotmail.com

Abstract

Beyrichitine ammonoids of NV Nevada reveal a high taxonomic diversity of Anisian (Middle Triassic). This diversity is, however, in contrast to their relatively low morphologic disparity. Depending on the exact definition, morphologic disparity of a data set is a

direct consequence of the sum of all ontogenetic changes. In the past, however, the interplay of both morphological processes has only rarely been addressed.

Using geometric morphometric methods, this study aims at a quantification of allometric processes and the morphologic disparity of beyrichitine ammonoids. The multivariate statistical analysis revealed that morphologic disparity, intraspecific variation respectively, within and between the studied species seems to be the result of deviations in the ontogenetic allometric growth pattern (i.e. heterochrony). During deposition of the studied stratigraphic sequence, a general progressive pedomorphism (juvenilization) was observed. The intraspecific variability pattern coincides with the total morphologic disparity of the analyzed species, which suggests that intraspecific variability facilitated morphologic disparity. The comparison of ontogenetic allometric patterns and changes in intraspecific variation and morphologic disparity are likely to refine our understanding of the intrinsic factors influencing the speciation of this group.

Keywords: Geometric morphometrics, heterochrony, allometry, ontogeny, morphologic disparity, morphospace, Beyrichitinae, Anisian, Nevada

Introduction

Analysis of morphology and ontogeny are the source for evolutionary and developmental studies in deep time. Since the late 20th century, developmental concepts such as heterochrony [e.g., 1, 2-6] and morphological disparity and intraspecific variation [e.g., 7, 8-10] have proven to be an invaluable source of information complementing taxonomic approaches [9, 11, 12] and enriching our knowledge of evolutionary dynamics [7, 11-

14]. Morphological disparity and intraspecific variation can be regarded on a hierarchy level. Morphological disparity is the quantification of morphological variation among species and higher taxa considers the variation within species [15]. If disparity is regarded as differences between ontogenetic end points, it can be argued that the total amount of disparity is a direct consequence of heterochrony *sensu* Alberch 1979 [13]. Knowledge of the ontogenetic trajectories is therefore a prerequisite for enlarging our understanding of macroevolutionary development [11]. However, despite their close relationship, morphologic disparity patterns have been relatively rarely addressed in the context of heterochronic analyses [e.g., 9, 13, 16-20].

In general, heterochrony is defined as change in timing (age) or rate (size) of development relative to the ancestor [21]. However, changes in rate and timing of ontogenetic events can, by definition, only be determined where the age of compared individuals is known [2; chap. 2]. Particularly in paleontology, exact growth rates are often not known [22, 23]. In ammonoids the accretionary growth with preservation of previous chambers, adds a relative time component to the analysis (i.e. the more whorls an individual has, the older it must be). Here it was assumed that the analyzed species have similar coiling rates (i.e., the individual species develop the same number of whorls in the course of their life). Where growth rate is similar between two groups, allometric relationships may reflect true heterochronies [2; chap. 2]. In general, ammonoids are ideal model organisms to study ontogenetic change, intraspecific variability, and macroevolutionary patterns [24-27]. That is reasoned in their wide paleogeographic distribution, high preservation potential and high taxonomic diversity and morphological disparity [28].

Previous research has shown that late Anisian (Middle Triassic) ammonoid assemblages of the Fossil Hill Member in NV Nevada are well suited as morphological case studies [12]. The ammonoids are very abundant and well preserved almost throughout the member. In addition, the continuous thin-layered calcareous successions were deposited in a rather stable and calm paleoenvironment [29]. During the depositional period of the Fossil Hill Member no major paleoenvironmental shifts were detected. Therefore, the sequences allow to trace morphological change of the species on a high-resolution stratigraphical scale.

Previous geometric morphometric analyses on the Anisian family Ceratitidae Mojsisovics, 1879 of Nevada revealed that members of this group cover a wide range of taxonomic diversity, which, however, is associated with rather low levels of morphologic disparity [12]. Quantification of morphological disparity, intraspecific variation and ontogenetic allometry would provide further insight into the evolutionary history of these species. However, by definition, phylogenetic information is crucial in determining heterochronic changes among taxa [2; chap. 2]. To our knowledge, for ceratitid ammonoids there is no phylogenetic framework available. For the analyses herein, we therefore focused on more closely related species of the subfamily Beyrichitinae Spath, 1934. The continuous successive stratigraphic sections of the Fossil Hill Member of NV Nevada allow to trace the development of individual species of this subfamily.

In this study, we used a landmark-based geometric morphometric approach including a suite of multivariate statistical tests to study morphological change through ontogeny of beyrichitine ammonoids. This is done in order to analyze heterochronic processes and compare the intra- and interspecific developmental patterns of these species. First, we analyzed ontogenetic allometric trajectories and compared the individual pattern to

each other at the species level. Furthermore, we investigated whether intra- and interspecific variability patterns of whorl shape can be noticed among all studied species. The analysis of such patterns might reveal important evolutionary mechanisms in the diversification of this clade. In a second step, we contrasted the concept of heterochrony, intraspecific variation and morphologic disparity in order to assess their interlinkage.

Material & methods

Fossil material

For this study 46 specimens representing a total of eight species of the genera *Gymnotoceras* Hyatt 1877, *Frechites* Smith 1932 and *Parafrechites* Silberling & Nichols 1982 were analyzed. These three genera all belong to the subfamily Beyrichitinae Spath, 1934. The selected species are the most characteristic ammonoids of the late Anisian Fossil Hill Member of NW Nevada, USA. All specimens were collected at the Fossil Hill of the Humboldt Range and in the Muller Canyon of the Augusta Mountains (Pershing County), north-western Nevada, USA. The material is stored in the Geosciences Collection of the University of Bremen (GSUB), Germany. More information on the meta data of the fossil material (i.e. geological framework, biostratigraphy, morphologic comparison, data acquisition) can be found in [12].

Members of the three genera in focus are morphologically similar [12, figs. 3–5]. The studied species belong to the discoidal morphospace and mainly differ through characteristic ways of ventral arching and some gradual differences in ribbing (Fig. 1). Bischof, Schlüter (12) have shown that all species follow the same ontogenetic pathway from very depressed to compressed whorls. However, while some species complete the entire

path, some stop their development at an earlier ontogenetic stage. This study therefore examines the closely related species in more detail and quantifies the allometric differences between them.

Figure 1. Cross sections of the analyzed ammonoid species of the late Anisian Fossil Hill Member of NW Nevada, USA; all × 1.5.

(A) Frechites nevadanus, GSUB C12377. (B) Frechites nevadanus, GSUB C13250. (C) Gymnotoceras weitschati, GSUB C14190. (D) Gymnotoceras blakei, GSUB C12243. (E) Gymnotoceras mimetus, GSUB C13814. (F) Gymnotoceras rotelliformis, GSUB 11594. (G) Parafrechites dunni, GSUB C9356. (H) Parafrechites meeki, GSUB C12536.

Data acquisition

The underlying shape data of this study represents an excerpt of the data of Bischof, Schlüter (12). The landmarks were retrieved using tpsDig2 v.2.31 [35]. Every half whorl is a separate configuration, which is represented by 16 landmarks (Fig. 2). The set of landmarks consists of two unpaired (1, 2) and seven pairs of landmarks (3–16), of which eight are sliding semi-landmarks. To omit missing values in subsequent analyses, the data set was limited to 11 half volutions (i.e., half whorl or growth stage number 5.5). Therefore, every specimen is represented by a total of 176 landmarks (16 landmarks on 11 half whorls).

Figure 2. Digitized sketch of high-precision cross-section of an ammonoid whorl with position of landmarks.

Filled crosses: fixed landmarks; empty crosses: sliding landmarks; black numbers: number of landmark. Definition of fixed landmarks: 1) venter of preceding whorl; 2) venter of whorl; 3 and

4) ventral shoulder or point of highest curvature; 5 and 6) maximum width; 7 and 8) Umbilical seam.

Geometric morphometric analysis

The exact appearance of any morphospace depends on the shapes which are being analyzed. Therefore, only the raw data of Bischof, Schlüter (12) were used and the basic geometric morphometric analysis was repeated as briefly summarized below in this sub-section. The adapted R script and the landmark data are provided in supporting information S1 and S2 files. All geometric morphometric analyses were done using the R software v 3.6.3. [R Core36] including the R packages *Morpho* v2.8 [37], *geomorph* v3.3.1. [38] and *RRPP* v0.6.0 [39, 40].

In a first step the 2D landmark coordinates were subjected to a full generalized Procrustes alignment (GPA) using the *Morpho*::*procSym* function. The full Procrustes fit standardizes size, orientation and position, leaving only the Procrustes shape coordinates [41]. Subsequently, the R function *geomorph*::*combine.subsets* was used to standardize all configurations of all growth stages to unit centroid size ($log_{10}CS$; un-weighted Procrustes shapes). Centroid size is regarded as a proxy for the size of the whorls and equals the square root of the summed squared distances of each landmark from the centroid of the landmark configuration before the GPA [3]. For the calculation of the two-dimensional morphospace we then ran a principal component analysis (PCA) on the aligned un-weighted Procrustes shape coordinates.

To illustrate morphologic variation an ontogenetic trajectory space and a developmental morphospace were calculated. In an ontogenetic trajectory space, the ontogenetic trajectory of every individual is reduced to a single data point [12, formerly called

ontogenetic morphospaces, 42]. The difference of allometric spaces *sensu* Gerber, Eble (11) to trajectory spaces is that the first use allometric trajectories of the size-shape space instead of ontogenetic trajectories. Whereas ontogenetic trajectory spaces are a tool to examine if the ontogenetic pathways of individuals differ, developmental morphospaces *sensu* [18, p. 40] describe how trajectories vary (chapter 4.2 and 4.3 of this thesis, formerly called ontogenetic morphospaces). Developmental morphospaces *sensu* Eble (18, p. 40) directly reflect developmental processes. In the context of this study this means that every individual dot in the developmental morphospace is represented by a specific ontogenetic stage (i.e. half whorl) of an individual [12]. The extreme shapes of the developmental morphospace, were calculated with the R function *GeometricMorphometricsMix*::*reversePCA* [43] and for the computation of the thin-plate spline deformation grids the R function geomorph::plotRefToTarget was used. All scatterplots were drawn with the R package ggplot2 [44].

Ontogenetic allometry

Depending on the scientific context, there are various, closely related biological and evolutionary developmental (evo-devo) terms that can be associated to studies of the size-shape space: Allometry [e.g. 11, 45, 46], allometric space [e.g. 11, 14] or simply heterochrony [e.g. 2, 4, 21]. To unravel patterns in the ontogenetic development of individuals and the phylogenetic variation between taxa, the size-shape relationship was analyzed here. In this study the allometric space was calculated with a regression analysis of the log-transformed size on the values of the first primary component (PC1) of each species (PCshape ~ $\log_{10}$CS*Group) using the R function *geomorph::procD.lm* with 999 iterations and Sum of Squares type I. To quantify the relationship between Procrustes shape

variables and a predictor (here log-transformed centroid size) the function fits a linear model to the Procrustes data and creates a size-shape space. A significant association between size and shape for particular species rejects the null hypothesis of isometry (no change in shape during growth) and reveals the influence of allometry.

Quantification of allometric trajectories

To quantify interspecific and intergeneric allometric relationships, we performed a homogeneity of slopes (HOS; R function *RRPP*::*pairwise*) test and a phenotypic trajectory analysis (R function *RRPP*::*trajectory.analysis* with 999 iterations). Both functions test for differences in the slope angle and length of the shape-size relationship to quantify the amount of shape variation, which is explained by size. More details on these procedures can be found in Esquerré, Sherratt (47, p. 2832).

Allometry is associated with changes during growth with age by definition. Since the age of fossil specimens can only hardly be determined, size and whorl number are used here as a proxy for age. Whereas the HOS test regards size as a continuous variable (i.e. $\log_{10}$size), the *trajectory.analysis* function depends on at least one categorical interaction variable (i.e. number of whorl) as a proxy for size. Therefore, the above computed allometric model (PCshape ~ $\log_{10}$CS*Group) was used in the HOS test and in the trajectory analysis a linear model including the whorl number (PCshape ~ Group * WhorlStage) was used. In general, differences in slope angles and maximum centroid size can be the result of heterochronic processes. For the terminology for heterochrony we follow the concept of [4] which is nicely illustrated in [21].

Morphologic disparity and intraspecific variation

To examine how intraspecific variability and morphological disparity changes through the development of individuals, we used the R function *geomorph::morphol.disparity*. Intraspecific variance is calculated in an analogous manner to morphological disparity [3]. The function calculates absolute differences in Procrustes variances of specified groups and tests for pairwise differences in Procrustes variances between these groups while accounting for group size. The statistical significance of the calculated Procrustes variances between the different growth stages was assessed using a randomized residual permutation test with 999 iterations.

It is very important to note that only discontinuous predictors (i.e. whorls) can be used here. In case a continuous predictor such as $log_{10}CS$ is used, the function *morphol.disparity* uses the overall mean of all configurations.

Results

Ontogenetic and developmental morphospaces

The ontogenetic (Fig. 3) and developmental (Fig. 4) morphospace of beyrichitine ammonoids do not substantially differ from the ceratitid morphospaces that were described in Bischof, Schlüter (12). The first three components (PCs) of the Principal Component Analysis (PCA) on the data set with joint configurations (i.e. ontogenetic trajectory spaces, Fig. 3) account for 63.5 % (PC_1 = 43.7 %, PC_2 = 15.6 %, PC_3 = 4.2 %) of the total variation. Since most of the variation is explained by the first component, the placement of the species along PC1 is the most important characteristic. Whereas the genus *Frechites* occupies the left side (low PC 1-values), in the central and right part of the dia-

gram *Gymnotoceras* and *Parafrechites* intermingle to some extent. This distribution is a first indication that the ontogenetic trajectories have taxonomic significance.

Figure 3. PCA plots of ontogenetic trajectory space of all species of (A) Principal Component 1 and 2; (B) Principal component 1 and 3.

The developmental morphospace (Fig. 4) shows the same three basic ontogenetic phases as described in Bischof, Schlüter (12): (1) Earliest whorls are flattened and occupy the lower left quadrant (negative PC1 and PC2 values; Fig. 5 A); (2) the depressed whorls of juveniles cover the central area of plot (PC1 = 0; PC2 slightly positive; Fig. 5 B); (3) adults have more compressed and stout whorls, right side of the diagram (positive PC1 and negative PC2 values; Fig. 5 C). The first three components of the PCA of all growth stages of the specimens as separate configurations (i.e. developmental morphospace) account for 93.8 % (PC_1 = 80.1 %, PC_2 = 10.7 %, PC_3 = 3.0 %) of the total variation.

Figure 4. Developmental morphospace with PCA of un-weighted Procrustes shape variables.

Point size refers to growth stage. Deformation grids show the transformation of the mean shape to the modeled shapes of the extreme values for PC1 and PC2.

Figure 5. Mean shapes of whorl stages.

(A) Whorl stage 0.5, (B) whorl stage 3.0 and (C) whorl stage 5.5.

Since the herein studied species show Type A and Type B ontogenetic trajectories (for a discussion on types see Bischof et al. 2021), there are two extreme adult shapes (Fig. 6): Type A) rather depressed, stout conches that do not overlap much the preceding whorl and are associated with much shorter ontogenetic trajectories (*F. nevadanus*, *F. occidentalis*, *P. meeki)*, and Type B) compressed conches with a more pronounced venter and a higher degree of overlap with the preceding whorl (*G. blakei*, *G. mimetus*, *G. rotelliformis*, *G. weitschati*, *P. dunni*).

Figure 6. TPS spline of mean shape of whorl 5.5 of all species in this study (grey) plotted against the mean shape of whorl 5.5 of the respective species (black).

Examining ontogenetic allometry: Regression model

Whorl shape is influenced by the centroid size, the group (species and/or genus) and the interaction of the two (R function *geomorph::procD.lm*; Table 1). This indicates that there is an ontogenetic allometric (and not isometric) pattern present and the allometric slopes differ amongst species and genus. The full model (PCshape ~ $\log_{10}$CS * species; Table 1) describes the shape significantly better than the reduced model (PCshape ~ $\log_{10}$CS; supporting information S3 file). Therefore, the full model was used for further analysis.

Table 1. Analysis of variance table obtained from the Procrustes ANOVA (R function RRPP::lm.rpp) of the full model (PCshape~ log10CS *group) on the species and genus level.

Df: Degrees of freedom; SS: Sum of squares; MS: mean squares; R^2: coefficient of determination; Z:

Effect sizes (Z) based on F distributions; Pr(>F): p-value of F statistics.

Species	Df	SS	MS	R^2	F	Z	Pr(>F)
**log.size**	1	0.98264	0.98264	0.70889	1463.5398	7.2528	0.001
**species**	7	0.02765	0.00395	0.01995	5.8834	6.1831	0.001
**log.size:species**	7	0.04689	0.00670	0.03383	9.9769	7.7669	0.001
**Residuals**	490	0.32899	0.00067	0.23734			
**Total**	505	1.38617					

Genus	Df	SS	MS	R^2	F	Z	Pr(>F)
**log.size**	1	0.98264	0.98264	0.70889	1401.3677	7.2141	0.001
**genus**	2	0.01675	0.00837	0.01208	11.9416	4.9199	0.001
**log.size:genus**	2	0.03619	0.01809	0.02611	25.8034	6.4041	0.001
**Residuals**	500	0.3506	0.0007	0.25293			
**Total**	505	1.38617					

The fitted values of the linear regression model were plotted against the $\log_{10}$CS (Fig. 7). Differences in slopes of the ontogenetic allometric trajectories are caused by changes in ontogenetic allometric patterns. There is not much variation in shape of whorl 0.5 between the individual species (Figs. 5, 7), but the range of different shapes of whorl 5.5 is more complex (Figs. 6, 7). The species *G. weitschati* displays a much smaller y-intercept (Fig. 7) than all other species. However, with three specimens this species had the smallest sample size (Fig. 4) and therefore also less statistical power.

Figure 7. Ontogenetic allometric trajectories with fitted values of the linear regression model plotted against log_{10}CS. Point size refers to growth stage.

TPS splines of Procrustes shapes for extreme regression values against the mean shapes of respective growth stage. The values of the y-intercept and the slopes of all species can be found in Table 2.

Table 2. Intercept and slopes of linear model of fitted shape values on centroid size.

Stratigraphic order from 1=old to 8=young.

**Species**	**Ontog. Type**	**Stratigraphic order**	**y-Intercept**	**Regression slope**
G. weitschati	B	1	-0.2914	0.05211
G. mimetus	B	2	-0.2852	0.04963
G. rotelliformis	B	3	-0.2626	0.04529
G. blakei	B	4	-0.2374	0.04107
P. dunni	B	7	-0.2247	0.039
P. meeki	A	6	-0.2119	0.03603
F. occidentalis	A	8	-0.2042	0.03372
F. nevadanus	A	5	-0.1867	0.03175

Evaluation and quantification of allometric slopes

Allometric slopes differ among species (Fig. 7) and genera as well. For the statistical quantification of the allometric trajectories an allometric trajectory analysis (R function *RRPP::trajectory.analysis*) and a homogeneity of slopes test (HOS; R function

RRPP::pairwise) were performed. Pairwise comparison of allometric trajectories revealed that all genera and most species have significantly different pairwise slopes (direction of shape change) in at least one of the two test procedures (see supporting information S4 file). Three species pairs did not reveal statistically significant slopes in either of the two tests (*G. blakei-P. dunni*, *G. weitschati-G. mimetus* and *G. weitschati-G. rotelliformis*). The members of the non-significant pairs all belong to type B.

Whereas the minimal centroid size does not vary much between the individual species, there is more variability in later ontogenetic stages. In general type A species attain smaller maximum (centroid) sizes than type B species. Accordingly, the prevailing heterochronic process from type B to type A can be regarded as a neoteny (pedomorphosis).

Intraspecific variation and morphological disparity during ontogeny

A general pattern in the levels of intraspecific Procrustes variance (Fig. 8 A) among the whorls can be observed in all investigated species: a faint decrease of relative variability occurs after the first whorl (0.5), with a general slight increase after whorl stage 1.5 or 2.0. The taxa *F. nevadanus*, *F. occidentalis* and *P. meeki*, have a relatively homogenous pattern with no significant differences among the whorls in the levels of Procrustes variance. In *G. weitschati*, *G. mimetus, G. blakei*, *G. rotelliformis* and *P. dunni*, in contrast, the Procrustes variance of whorl 5.5 is approximately doubled to tripled compared to previous whorls (see. Fig. 8 A for visual comparison, supporting information S5 file for data). Whereas the Procrustes variance of whorls 0 to 5.0 of *G. blakei*, *G. rotelliformis* and *P. dunni*, are again relatively homogenous, as in above mentioned species, *G. mimetus*

and G. weitschati are the only species that have a second significant minima of variance between the whorl stage 3.0 and 5.5.

Figure 8. Amount of Procrustes variance of intraspecific variation and morphologic disparity of whorl stages and species.

(A) Intraspecific variability patterns broken down by whorl stage. Contribution of individual whorls of species to intraspecific variation. The x axis shows the growth stages from 0.5 to 5.5. (B) Total Procrustes variance (i.e. morphological disparity) of all species at specific growth stages. The x axis shows the growth stages from 0.5 to 5.5. (C) Contribution of species to overall intraspecific variation. Species are ordered in stratigraphic order from left-old to right-young.

The total variance of the respective whorls (Fig. 8 B) of the investigated species is relatively homogenous among whorls 0.5–5, with a relatively small drop from whorl 0.5 to whorl 1.5. The variance of following whorls is generally increased again, though, admittedly, no significant differences among whorls 0.5 – whorl 5 can be reported; only whorl 5.5 with the most elevated level of Procrustes variance differs significantly to previous whorls.

The total intraspecific variation (Fig. 8 C) in stratigraphic order shows a downward trend from old to young. Especially within the evolutionary line of the genus *Gymnotoceras* the intraspecific variation is constantly decreasing.

Discussion

In this study, ontogenetic allometric trajectories and intra- and interspecific variability patterns of ammonoids were quantified for the first time using geometric morphometric methods. The methods used represents an extension of the methods previ-

ously introduced by Bischof, Schlüter (12). The analysis of the evolutionary patterns revealed that the analyzed species do not really differ through morphological shapes that are developed *per se*, but rather through their individual developmental rates, i.e. heterochrony (Figs. 4, 8). This is confirmed by differences in ontogenetic (Fig. 3) and allometric ontogenetic trajectories (Fig. 7) among species. The ontogenetic development of the species in focus are characterized by several changes in intraspecific variability (Fig. 8) and developmental rate or "timing" of whorl outline (Fig. 7; Table 1). These findings go in line with previous research on ceratitid ammonoids by Bischof, Schlüter (12).

Patterns in ontogenetic allometry

The ontogenetic development of the species and genera in focus are characterized by changes in developmental rate or "timing" of whorl shape (Fig. 7; Table 1), which manifests itself in an almost constant decrease of the allometric slope in stratigraphic order (Table 2). In general, adult representatives of stratigraphically younger species resemble juveniles of stratigraphically older individuals, i.e. pedomorphosis or juvenilization [2, p. 14, 4, 5, p. 209].

Due to their small size (+/- 0.4 mm) the earliest whorls are more difficult to measure than later and larger ontogenetic stages. However, more importantly, this large difference implies also that the relative measurement error associated with the earliest (smaller) ontogenetic stages is much larger [12]. Such measurement inaccuracies have a greater influence on the y-intercept (more strongly influenced by shape at origin) than on the slope of the allometric trajectories (reflects interplay of all shapes during ontogeny). Therefore, it is suggested that pre- and/or post-replacement of the allometric trajectories was induced by measurement inaccuracies. The full ontogenetic sequence of the

studied specimens, as already described by Bischof, Schlüter (12), includes the transition from flat to depressed to compressed whorls. Thereby the studied species can be divided in two main ontogenetic groups: B) longer trajectories that refer to an elongated adult whorl shape (*G. blakei*, *G. mimetus*, *G. rotelliformis*, *G. weitschati*, *P. dunni*), and A) truncated trajectories that are caused by "juvenilized" (pedomorphic), rather rounded and stout adult whorls (*F. nevadanus*, *F. occidentalis*, *P. meeki*). Regarding the quantification of allometric heterochrony the following three main statements can be made: (1) The pairwise difference in slope of the individual species and genera are statistically significant. (2) The evolutionary lineage of *Gymnotoceras* lineage is characterized by a progressive decrease in allometric slope, i.e. neoteny (pedomorphosis). (3) The smaller allometric slope of Type A species compared to Type B species is indicative for neoteny (resulting in pedomorphosis).

Paleobiologists argue that certain heterochronic changes would be adaptively favored in particular environments [48]. Thereby, more stable environments can rather be associated with slowed down growth of the paedomorphic development (Neoteny) [2, chap. 4, 5, chap. 8]. The relatively calm and stable paleoenvironment under which the successions of the Fossil Hill Member were deposited [29] do not challenge this picture.

Intraspecific variability and morphological disparity

Two unequivocal patterns of intraspecific variability during ontogeny can be recognized among the studied species (Fig. 8). The first pattern is characterized by homogeneous levels of Procrustes variance among the whorls and can be detected in *F. nevadanus*, *F. occidentalis* and *P. meeki* (ontogenetic type A). The ontogenetic trajectories of these species are shorter (Fig. 4), i.e. reflect pedomorphic development. The second pat-

tern shows a significantly increased level of Procrustes variance in the last whorl (whorl 5.5). The latter pattern is particularly seen in ontogenetic type B species: *G. weitschati*, G. *blakei*, *G. rotelliformis*, *G. mimetus* and *P. dunni*. Similar developmental patterns were detected by Gerber, Neige (49; tab. 2; fig. 6) that described significant higher levels of variance of adult hildoceratid ammonoids than their corresponding juveniles. By applying a traditional morphometric approach, Hoffmann, Weinkauf (50) studied intraspecific variation during the ontogeny in ammonoids, resulting in no clear patterns in variation. The differences in study design hinder a close comparison of their study with ours.

In general, there is no clear-cut pattern in the variability during ontogeny in ammonoids. Studies report ontogenies for instance with a decrease in variability from oldest to youngest stages and vice versa [13, 51]. The intraspecific variability patterns of the studied species can be explained by their ontogenetic developmental grouping rather than by their taxonomic assignment. Particularly within the evolutionary line of the genus *Gymnotoceras* the total Procrustes variance (Fig. 8 C) is characterized by a progressive decrease in stratigraphic order (from old to young).

In case of the studied material elevated levels of intraspecific variation coincide with the occurrence of the three "extreme" shapes (flat – depressed – compressed; Fig. 5). When transitions between developmental stages are accompanied by abrupt changes [critical points or "Knickpunkte"; 52, 53], developmental disparity patterns are likely to be polyphasic as well [49]. It is remarkable that in the intraspecific variability of particularly the ontogenetic type B group (Fig. 8A; *G. weitschati*, *G. blakei*, *G. rotelliformis*, *G. mimetus* and *P. dunni*) a pattern is revealed which coincides with the total interspecific morphologic disparity of the analyzed species (Fig 8B). The variance of the latest whorl (5.5) is significantly elevated, in comparison to the remaining whorls, in either both,

morphologic disparity and intraspecific variability, patterns. Accordingly, the shape of whorl 5.5, which yields the highest disparity in the analyzed species, has also the highest level of intraspecific variability. This might indicate weakened developmental constraints that promote disparity in the shape of the latest whorl among the analyzed beyrichitine ammonoids. Similar observations were made in species of the Late Cretaceous echinoid *Micraster* [54].

Conclusion

Even though heterochronic processes are possible factors responsible for intraspecific variation and morphologic disparity, the interplay of heterochrony and morphological disparity has only rarely been addressed [13]. In this study heterochronic relationships and morphologic disparity of ammonoids were quantified using geometric morphometric methods on ontogenetic cross-sections for the first time. Comparisons of disparity, when combined with multivariate statistical analyses of ontogenetic allometric trajectories, help to quantitatively assess the role of development in shaping morphospace occupation and adult disparity [11].

The geometric morphometric analysis of this study revealed that intraspecific variability patterns of the studied species are only roughly linked to their taxonomic classification; rather it is explained by their individual ontogenetic developmental grouping. Therefore, intraspecific variation and morphologic disparity in beyrichitines seems to be the result of perturbations of the allometric growth pattern (i.e. heterochrony). There is evidence that more stable environments can generally rather be associated with slowed down growth of pedomorphic development, i.e. neoteny [2, chap. 4, 5, chap. 8]. This suggests that the rather calm and stable environment of the Fossil Hill Member [29] favored

pedomorphic processes, which is reflected by the continuously decreasing allometric slopes (Fig. 7) of the species studied here.

The comparison of ontogenetic allometry patterns and changes in morphologic disparity are likely to refine our understanding of the intrinsic factors influencing the speciation of this group. Even if the methods introduced herein might not supply the full causal explanation on the diversity and disparity patterns observed, they offer additional insights into the macroevolutionary processes in ammonoids. This study therefore underlines the importance of using quantitative multivariate analyses to properly assess the role of ontogenetic processes in shaping morphologic disparity across species.

Acknowledgements

We would like to thank D. Kuhlmann (now Messel, Germany) as former technician of the Geowissenschaftliche Sammlung Bremen for field assistance and mechanical preparation of the material collected by our working group. M. Krogmann (Bremen, Germany) is thanked for field assistance as well as his broad support in the artwork for this article. Our gratitude goes to D. Korn (Berlin) for scientific discussion and moral support during the design of this study. We are indebted to K. Boos for the introduction to the R software and further statistical support. P. Embree (Orangevale, CA, USA) is thanked for broad support, including organization of field-campaigns, scientific information and the permission to collect on his private property. Our gratitude goes to the reviewers…

References

1. McNamara KJ. Heterochrony. In: Briggs DEG, Crowther PR, editors. Oxford-London-Edinburgh-Boston-Melbourne: Blackwell; 1990. p. 111-9.
2. McKinney ML, McNamara KG. Heterochrony. The Evolution of Ontogeny. 1991:XXI+437.
3. Zelditch ML, Swiderski DL, Sheets HD. Geometric morphometrics for biologists: a primer. 2012:478.
4. Alberch P, Gould SJ, Oster GF, Wake DB. Size and shape in ontogeny and phylogeny. Paleobiology. 1979;5(3):296-317. PubMed Central PMCID: PMC1.
5. Gould SJ. Ontogeny and phylogeny. 1977:501.
6. Klingenberg CP. Heterochrony and allometry: the analysis of evolutionary change in ontogeny. Biological Reviews. 1998;73:79-123. PubMed Central PMCID: PMC1.
7. Foote M. The Evolution of Morphological Diversity. Annual Review of Ecology and Systematics. 1997;28(1):129-52. doi: 10.1146/annurev.ecolsys.28.1.129.
8. Ciampaglio CN, Kemp M, McShea DW. Detecting changes in morphospace occupation patterns in the fossil record: characterization and analysis of measures of disparity. Paleobiology. 2001;27(4):695-715.
9. Eble GJ. Contrasting evolutionary flexibility in sister groups: disparity and diversity in Mesozoic atelostomate echinoids. Paleobiology. 2000;26(1):56-79. PubMed Central PMCID: PMC2.
10. Gould SJ. The disparity of the Burgess Shale arthropod fauna and the limits of cladistic analysis: why we must strive to quantify morphospace. Paleobiology. 1991:411-23.

11. Gerber S, Eble GJ, Neige P. Allometric space and allometric disparity: a developmental perspective in the macroevolutionary analysis of morphological disparity. Evolution. 2008;62(6):1450-7. doi: 10.1111/j.1558-5646.2008.00370.x.
12. Bischof EA, Schlüter N, Korn D, Lehmann J. Ontogeny of highly variable ceratitid ammonoids from the Anisian (Middle Triassic). PeerJ. 2021;9:e10931.
13. McNamara KJ, McKinney ML. Heterochrony, disparity, and macroevolution. Paleobiology. 2005;31(2):17-26.
14. Gerber S. Comparing the differential filling of morphospace and allometric space through time: the morphological and developmental dynamics of Early Jurassic ammonoids. Paleobiology. 2011;37(3):369-82.
15. Hopkins MJ, Gerber S. Morphological disparity. Nuño de la Rosa L, Müller GB, editors Evolutionary Developmental Biology Springer International Publishing. 2017:1-12.
16. Eble GJ. The role of development in evolutionary radiations. In: McKinney M, Drake J, editors. Biodiversity dynamics: Turnover of populations, taxa, and communities1998. p. 132-61.
17. Eble GJ. Multivariate approaches to development and evolution. In: Minugh-Purvis N, McNamara KG, editors.: The Johns Hopkins University Press; 2002. p. 51-78.
18. Eble GJ. Developmental morphospaces and evolution. In: Crutchfield JP, Schuster PM, editors. Santa Fe Institute Studies on the Sciences of Complexity: Oxford University Press; 2003. p. 33-63.
19. Zelditch M, Sheets HD, Fink W. The ontogenetic dynamics of shape disparity. Paleobiology. 2003:139–56. doi: 10.1666/0094-8373(2003)029<0139:TODOSD>2.0.CO;2. PubMed Central PMCID: PMC5.

20. Crônier C. Morphological disparity and developmental patterning: contribution of phacopid trilobites. Palaeontology. 2013;56:1263-71.
21. McNamara KJ. Heterochrony: the evolution of development. Evolution: Education and Outreach. 2012;5(2):203-18.
22. Knauss MJ, Yacobucci MM. Geographic Information Systems technology as a morphometric tool for quantifying morphological variation in an ammonoid clade. Palaeontologia Electronica. 2014;17(1):1-27. PubMed Central PMCID: PMC2.
23. Lécuyer C, Bucher H. Stable isotope compositions of a late Jurassic ammonite shell: a record of seasonal surface water temperatures in the southern hemisphere? eEarth Discussions. 2006;1:1-19.
24. Seilacher A. Why are nautiloid and ammonite sutures so different ? Neues Jahrbuch für Geologie und Paläontologie, Abhandlungen. 1988;177(1):41-69.
25. Landman NH, Tanabe K, Shigeta Y. Ammonoid embryonic development. In: Landman NH, Tanabe K, Davis RA, editors. Topics in Geobiology. 13. New York: Plenum; 1996. p. 343-405.
26. Monnet C, De Baets K, Klug C. Parallel evolution controlled by adaptation and covariation in ammonoid cephalopods. BMC Evolutionary Biology. 2011;11 (115)(1):1-21. doi: 10.1186/1471-2148-11-115.
27. Bucher H, Landman NH, Klofak SM, Guex J. Mode and rate of growth in ammonoids. In: Landman NH, Tanabe K, Davis RA, editors. Topics in Geobiology. 13. New York: Plenum; 1996. p. 407-61.
28. Korn D. Quantification of ontogenetic allometry in ammonoids. Evolution & Development. 2012;14(6):501-14. doi: doi:10.1111/ede.12003.

29. Bischof EA. Ontogenetic and macroevolutionary patterns of Anisian (Middle Triassic) ammonoids from Nevada, USA [dissertation]: University of Bremen; 2021.
30. Mojsisovics Ev. Vorläufige kurze Übersicht der Ammoniten-Gattungen der mediterranen und juvavischen Trias. Verhandlungen der Kaiserlich-Königlichen Geologischen Reichsanstalt Wien. 1879;1879:133-43. PubMed Central PMCID: PMC1.
31. Spath LF. Catalogue of the fossil Cephalopoda in the British Museum (Natural History): Part IV The Ammonoidea of the Trias. 1934:521.
32. Hyatt A. Part I. Palæontology. In: Meek FB, editor. Report of the US Geological Exploration of the 40th Parallel. 4. 4/1. Washington: US Government Printing Office; 1877. p. 110-1.
33. Smith JP. Lower Triassic Ammonoids of North America. 1932:iv+199.
34. Silberling NJ, Nichols KM. Middle Triassic molluscan fossils of biostratigraphic significance from the Humboldt Range, northwestern Nevada. US Geological Survey Professional Paper. 1982. PubMed Central PMCID: PMC4.
35. Rohlf F. TPSDig2: A program for landmark development and analysis: Department of Ecology and Evolution, State University of New York; 2010 [cited Available at: http://life.bio.sunysb.edu/morph (accessed at January 30, 2018)].
36. Team RC. R: A Language and Environment for Statistical Computing. Version 3.6.3. Vienna, Austria: R Foundation for Statistical Computing; 2020.
37. Schlager S. Morpho and Rvcg – Shape Analysis in R: R-Packages for Geometric Morphometrics, Shape Analysis and Surface Manipulations. In: Zheng G, Li S, Székely G, editors. San Diego: Academic Press; 2017. p. 217-56.

38. Adams DC, Collyer ML, A. K. Geomorph: Software for geometric morphometric analyses. R package version 3.2.1. https://cran.r-project.org/package=geomorph. 2020.
39. Collyer ML, Adams DC. RRPP: An R package for fitting linear models to high-dimensional data using residual randomization. Methods in Ecology and Evolution. 2018;9(2):1772-9.
40. Collyer ML, Davis MA, Adams DC. Making heads or tails of combined landmark configurations in geometric morphometric data. Evolutionary Biology. 2020:1-13.
41. Hammer Ø, Harper D. Paleontological data analysis. 2005:368.
42. Bischof EA, Lehmann J. Ontogenetic analysis of Anisian (Middle Triassic) ptychitid ammonoids from Nevada, USA. Journal of Paleontology. 2020;94:829-51. doi: 10.1017/jpa.2020.25. PubMed Central PMCID: PMC6.
43. Fruciano C. GeometricMorphometricsMix: Miscellaneous functions useful for geometric morphometrics. R package version 0.0.7.9000. 2019.
44. Wickham H. ggplot2: elegant graphics for data analysis. New York: Springer; 2016.
45. Klingenberg CP. Multivariate allometry. In: F. ML, M. C, A. L, P. NGJ, E. SD, editors. New York: Plenum Press; 1996. p. 23-49.
46. Mitteroecker P, Gunz P, Windhager S, Schaefer K. A brief review of shape, form, and allometry in geometric morphometrics, with applications to human facial morphology. Hystrix, the Italian Journal of Mammalogy. 2013;24(1):59-66. PubMed Central PMCID: PMC1.
47. Esquerré D, Sherratt E, Keogh JS. Evolution of extreme ontogenetic allometric diversity and heterochrony in pythons, a clade of giant and dwarf snakes. Evolution. 2017;71(12):2829-44. PubMed Central PMCID: PMC1.

48. Yacobucci MM. Macroevolution and paleobiogeography of Jurassic-Cretaceous Ammonoids. In: Klug C, Korn D, De Baets K, Kruta I, Mapes RH, editors. Topics in Geobiology. 44. Dordrecht: Springer; 2015. p. 189-228.
49. Gerber S, Neige P, Eble GJ. Combining ontogenetic and evolutionary scales of morphological disparity: a study of early Jurassic ammonites. Evolution & development. 2007;9(5):472-82. PubMed Central PMCID: PMC3.
50. Hoffmann R, Weinkauf MFG, Wiedenroth K, Goeddertz P, De Baets K. Morphological disparity and ontogeny of the endemic heteromorph ammonite genus Aegocrioceras (Early Cretaceous, Hauterivian, NW-Germany). Palaeogeography, Palaeoclimatology, Palaeoecology. 2019;520:1-17. doi: https://doi.org/10.1016/j.palaeo.2019.01.020. PubMed Central PMCID: PMC4.
51. De Baets K, Bert D, Hoffmann R, Monnet C, Yacobucci MM, Klug C. Ammonoid intraspecific variability. In: Klug C, Korn D, De Baets K, Kruta I, Mapes RH, editors. Topics in Geobiology. 43. Dordrecht: Springer; 2015. p. 359-426.
52. Kullmann J, Scheuch J. Wachstums-Änderungen in der Ontogenese paläozoischer Ammonoideen. Lethaia. 1970;3:397-412.
53. Kant R, Kullmann J. "Knickpunkte" im allometrischen Wachstum von Cephalopoden-Gehäusen. Neues Jahrbuch für Geologie und Paläontologie, Abhandlungen. 1973;142(1):97-114. PubMed Central PMCID: PMC1.
54. Schlüter N, Wiese F. The variable echinoid *Micraster woodi* sp. nov. – Trait variability patterns in a taxonomic nightmare. Cretaceous Research. 2018;87:194-205. doi: https://doi.org/10.1016/j.cretres.2017.05.019.

Supporting information

S1 R script for Geometric Morphometric analysis.

S2 Landmark data of analyzed species (retrieved with tpsDig2).

S3 Table 1. Reduced Model (shape~ log10CS): Analysis of variance table obtained from the Procrustes ANOVA (R function RRPP::lm.rrpp) of the reduced model.

S3 Table 2. Results of comparison of reduced and full model: Analysis of variance table obtained from the Procrustes ANOVA (R function stats::anova).

S4 Table 1. Results of R function RRPP::trajectory.analysis on species level- Trajectory correlations from every permutation.

S4 Table 2. Results of R function RRPP::pairwise - Homogeneity of slopes test on species level.

S4 Table 3. Results of R function RRPP::trajectory.analysis on genus level- Trajectory correlations from every permutation.

S4 Table 4. Results of R function RRPP::pairwise - Homogeneity of slopes test on genus level.

S5 Table 1. Intraspecific variability patterns broken down by whorl stage (raw data for Fig. 8A).

S5 Table 2. Morphological disparity at specific growth stages (raw data for Fig. 8B).

S5 Table 3. S5 Table 3. Intraspecific variation of species (raw data for Fig. 8C).

Figure 1

Click here to access/download;Figure;Fig-1_Cross-sections_v1-1.tif

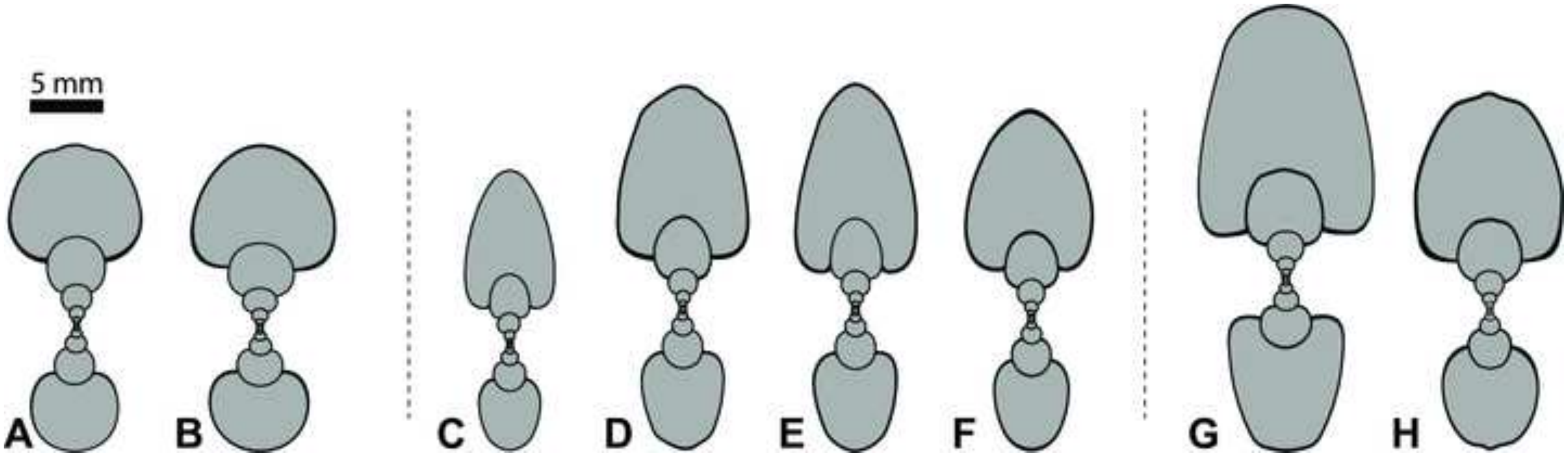

Figure 2

Click here to access/download;Figure;Fig-2_halfwhorl_v1-1.tif

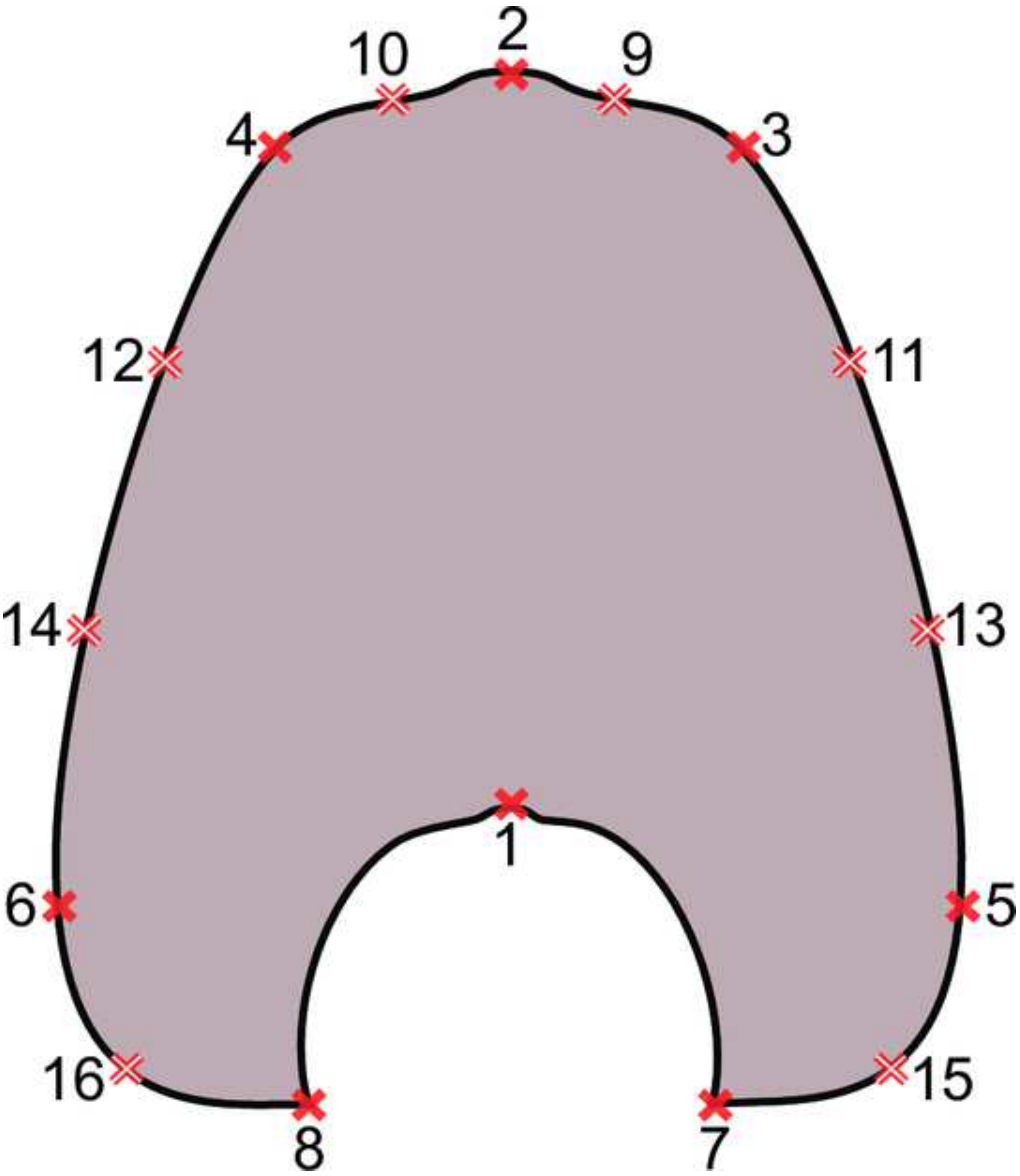

Figure 3

Click here to access/download;Figure;Fig-3_OntoTrajSpace.tif

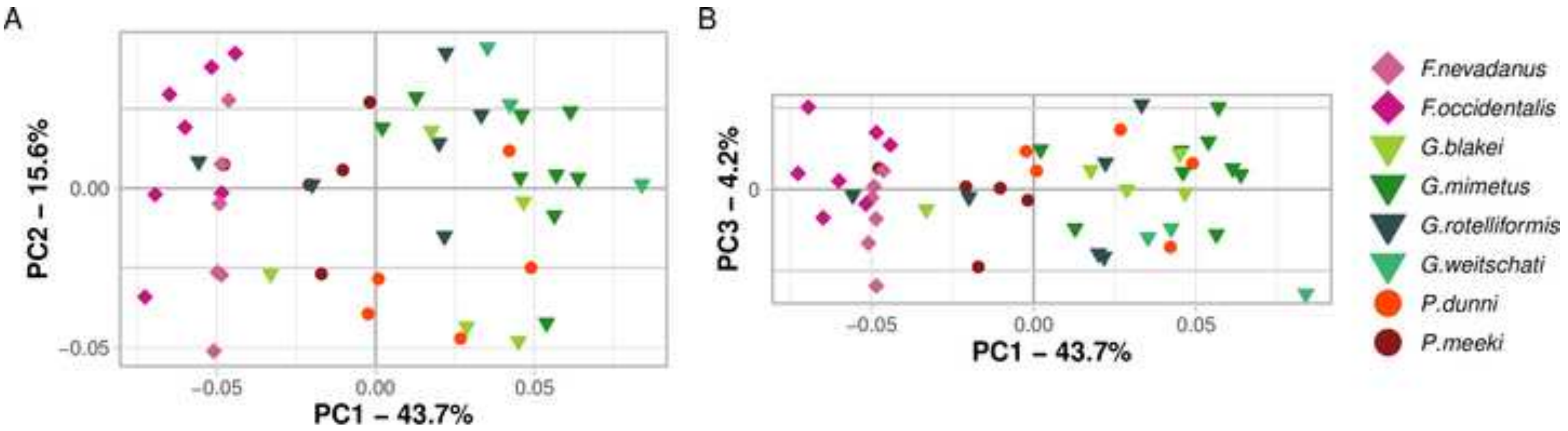

Click here to access/download;Figure;Fig-4_Deve_MS.tif

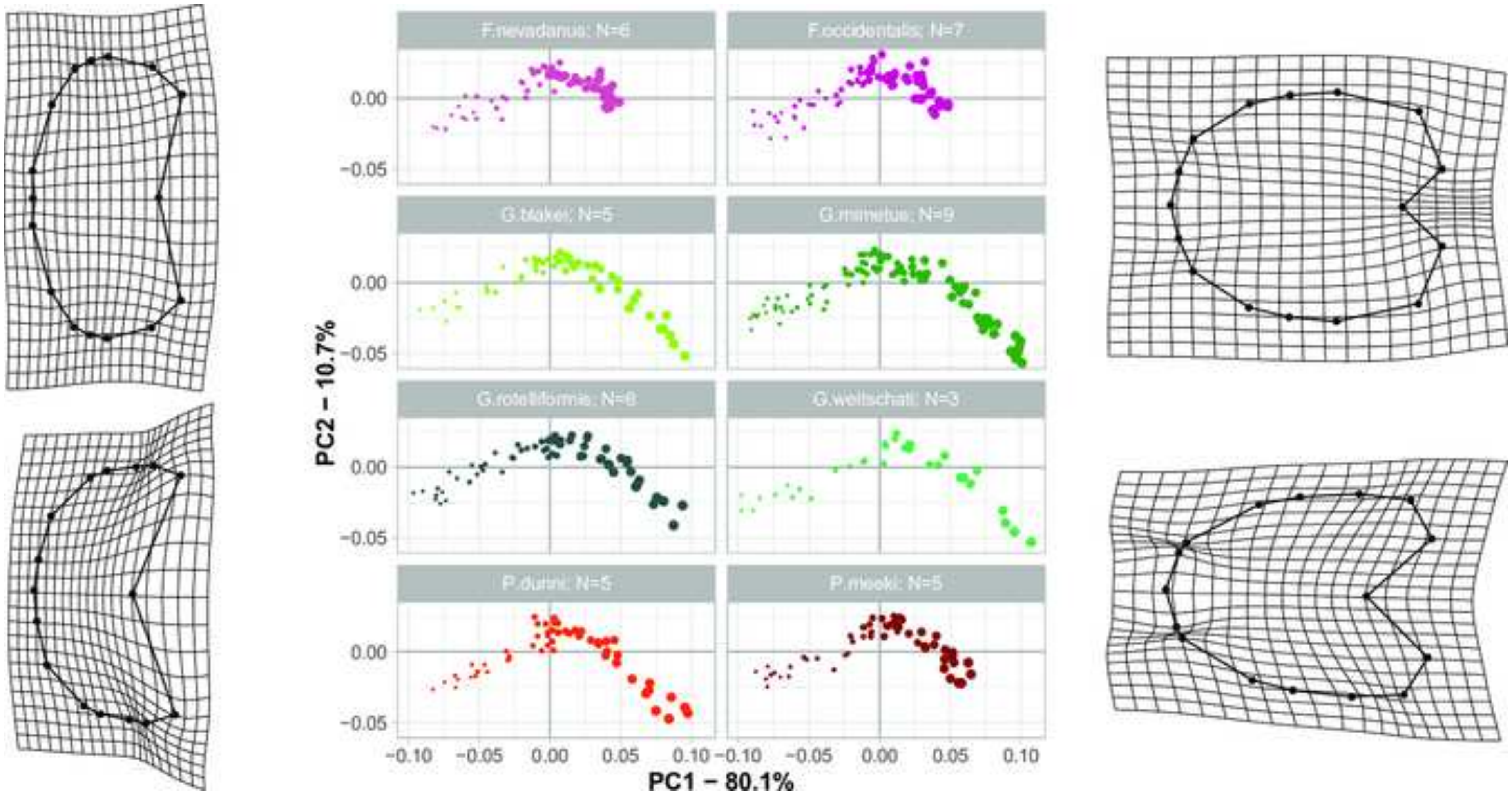

Figure 5

Click here to access/download;Figure;Fig-5_Mean3Shapes.tif

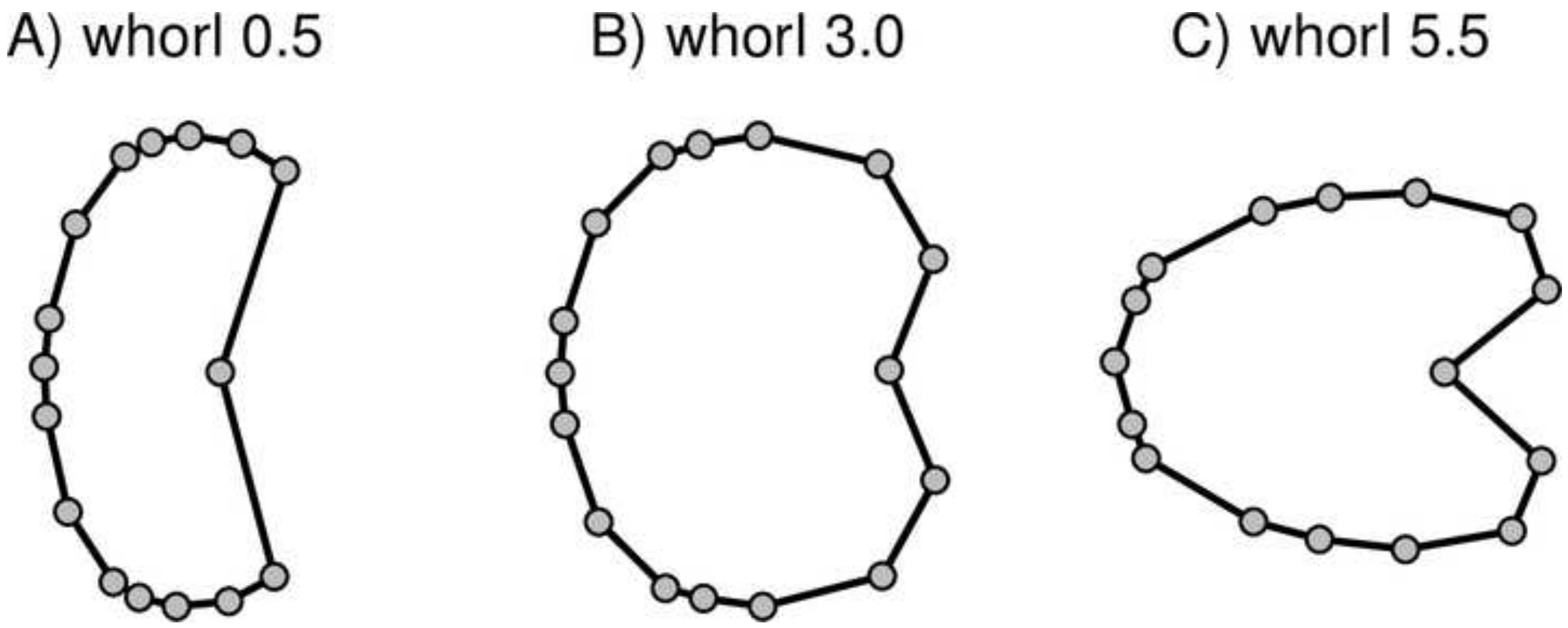

Figure 6

Click here to access/download;Figure;Fig-6_MeanShapes_5-5.tif

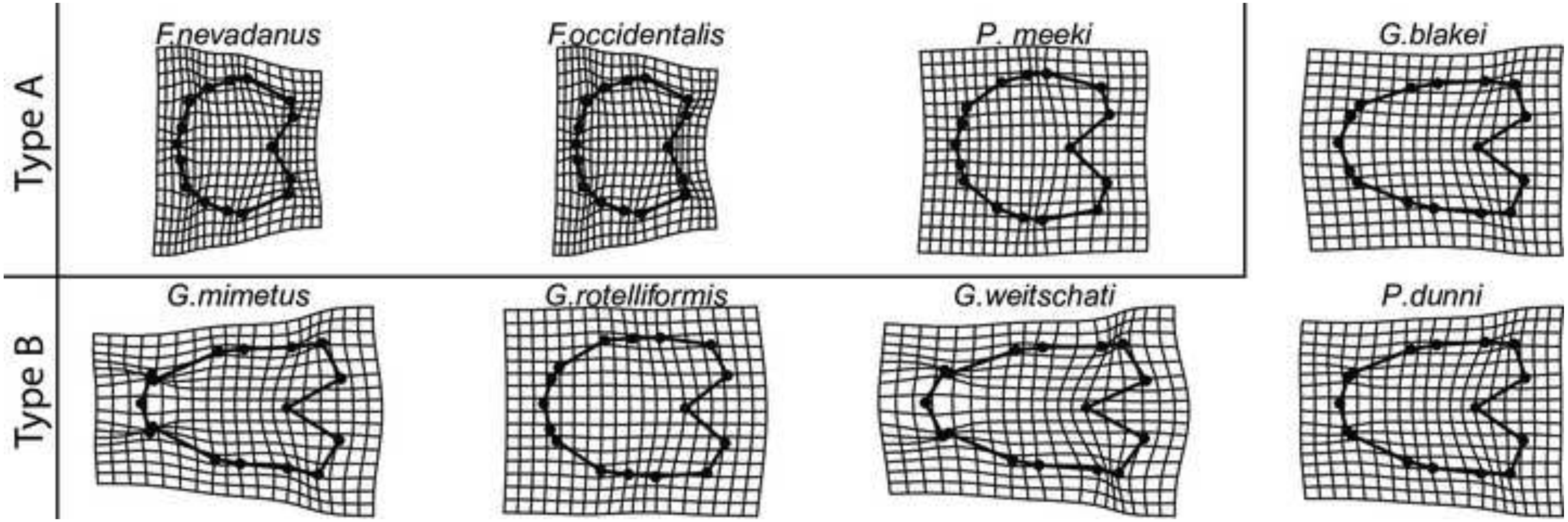

Figure 7

Click here to access/download;Figure;Fig-7_RegShapes_v1-1.tif

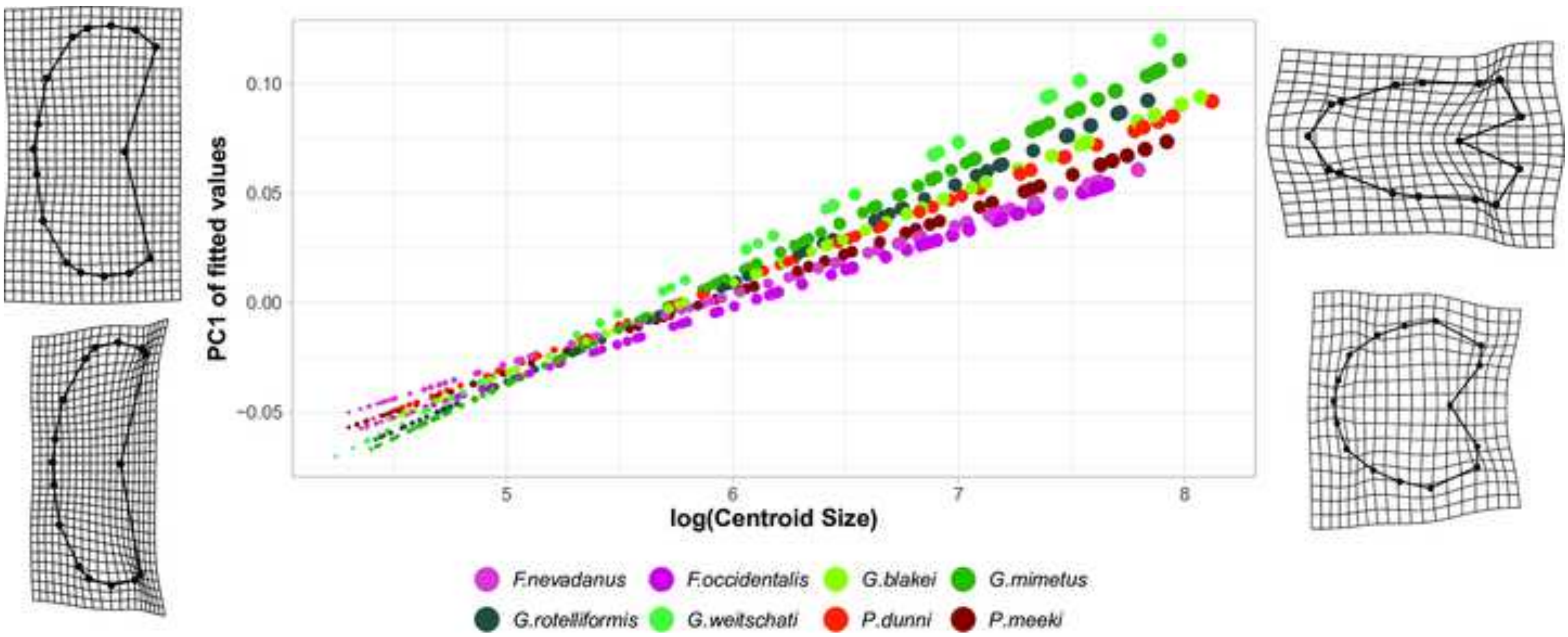

Figure 8

Click here to access/download;Figure;Fig-8_Disparity.tif

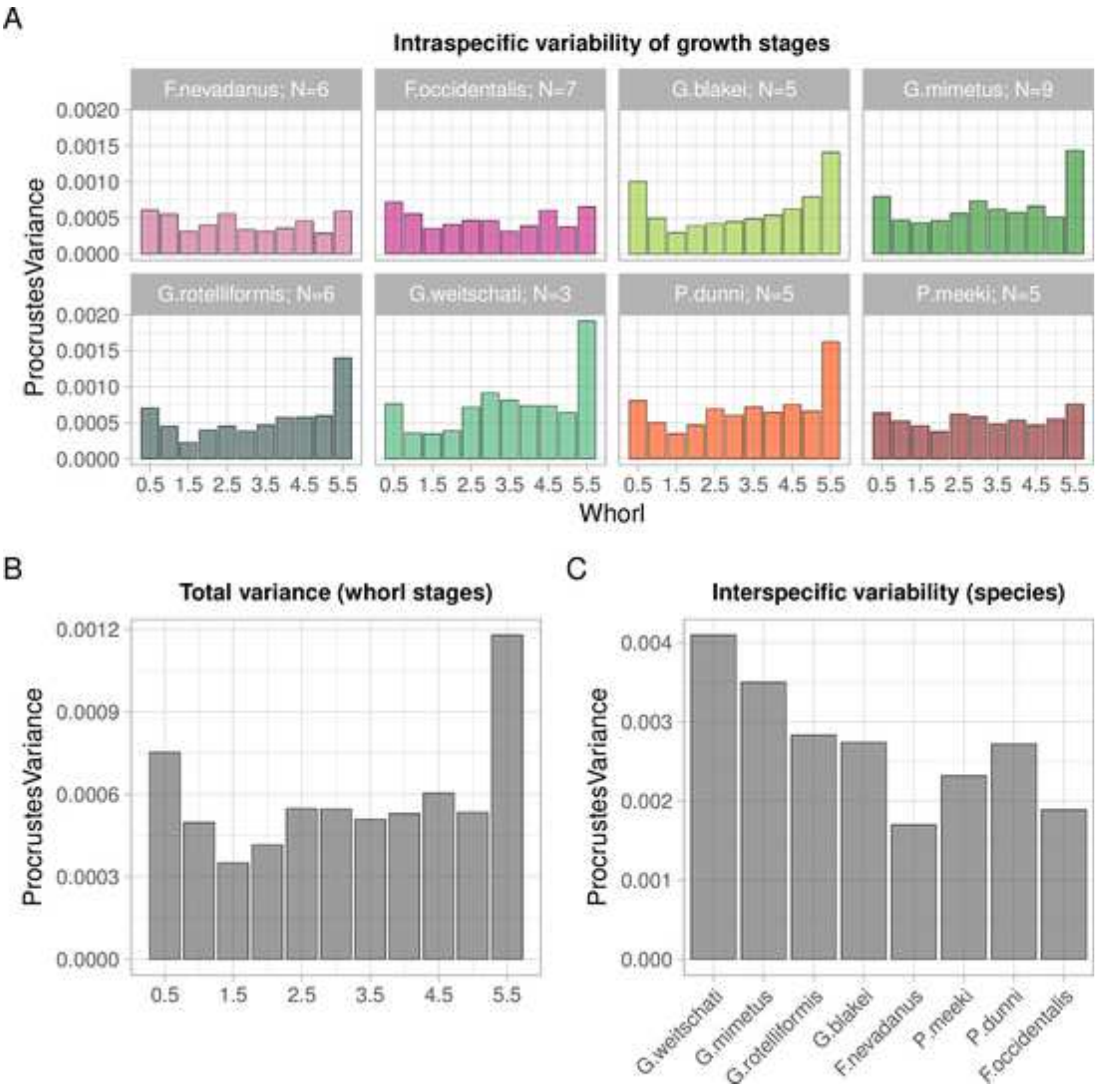

S1 R Script

Click here to access/download
Supporting Information
S1_Script_GMM_Beyrichitine.R

S2 Landmark Data

Click here to access/download
Supporting Information
S2_Data_GMM_Beyrichitine.TPS

S3 Results of regression analysis

Click here to access/download
Supporting Information
S3_SupportingInformation_Reg.xlsx

S4 Results of trajectory analysis

Click here to access/download
Supporting Information
S4_SupportingInformation_Traj-Anal_HOS.xlsx

S4 Results of analysis of Procrustes variance

Click here to access/download
Supporting Information
S5_SupportingInformation_ProcVar.xlsx
