# Supplementary material for: Geometric morphometric analysis of morphologic disparity, intraspecific variation and ontogenetic allometry of beyrichitine ammonoids
Source: PLoS One. 2022 Feb 10;17(2):e0263524. doi: 10.1371/journal.pone.0263524 (PMC8830730; doi:10.1371/journal.pone.0263524)
Supplement: Attachment — Submitted filename: Response to Reviewers.docx [file pone.0263524.s007.docx]

**Answers to Editor E-mail:**

**1. When submitting your revision, we need you to address these additional requirements.**

Answer: I checked and updated every formatting mistake I could find. I hope that my ms now fully meets PlosONEs expectation. A word template would make it much easier to format it correctly.

**2. In your Methods section, please provide additional information regarding the permits you obtained for the work. Please ensure you have included the full name of the authority that approved the field site access and, if no permits were required, a brief statement explaining why.**

Answer: I added the paragraph.

**3.Thank you for stating the following in your Competing Interests section:**

**“There are no competing interests.”**

**Please complete your Competing Interests on the online submission form to state any Competing Interests. If you have no competing interests, please state ""The authors have declared that no competing interests exist."", as detailed online in our guide for authors at http://journals.plos.org/plosone/s/submit-now**

**This information should be included in your cover letter; we will change the online submission form on your behalf.**

Answer: I included the sentence “There are no competing interests.” In the revised version of the cover letter.

**4. We note that you have stated that you will provide repository information for your data at acceptance. Should your manuscript be accepted for publication, we will hold it until you provide the relevant accession numbers or DOIs necessary to access your data. If you wish to make changes to your Data Availability statement, please describe these changes in your cover letter and we will update your Data Availability statement to reflect the information you provide.**

Answer: There must be a misunderstanding. I uploaded the R script and the data as Supporting Information, there is no additional data which I did not upload directly to PlosONE.

**Answers to comments in email from Marco Balini:**

**Comment: It would be interesting to compare the sutures line of the studied species, in order to test if the differences in the suture lines are consistent with pedomorphic relationship.**

Answer: I agree that it would be particularly interesting to extend the analysis with suture data. However, there are two main difficulties: 1) There are no suture lines visible at the fossil material at hand. 2) Before doing so it would be necessary to come up with a GMM approach to compare suture lines. That would be very interesting future work!

**Answers to comments in email PDF of Kenneth de Baets:**

**Comment email: I just would like to see a little bit more discussion on previous research in this direction as I feel this would place your work in even broader context, widen its scope and further highlight the importance and innovativeness of your approach.**

Answer: I included the suggested references (more detailed answers on individual cases below)

**1) The consideration of heterochrony and ontogenetic trajectories when comparing specimens within and between species: this approach has a long tradition particularly in ammonoid research (e.g., Michalsky 1890 and more formally by Schmidt 1926). Studying intraspecific variation throughout ontogeny and differences (or lack thereof) between species has also been studied by various authors (e.g., particularly in Paleozoic ammonoids: Korn and Vöhringer 2004; Korn and Klug 2007; Ebbighausen and Korn 2007; De Baets et al. 2013; but also, Mesozoic ammonoids: Dommergues and Marchand 1986; Gerber et al. 2007, 2008; Gerber 2011).**

Answer: Most of these studies were already mentioned in the introduction (right at the beginning). However, I updated the list of studies and included the ones that were not already part of the ms (in the introduction but also the discussion part).

**2) Heterochrony and the environment: you mainly cite McKinney to back up this association, which is classical work, but additional publications have been published particularly targeting ammonoids (e.g., Landman and Geyssant 1993; Gerber 2011). Also, I feel it would be more appropriate to cite the full chapter reference giving credit to author of individual chapters of McKinney 1991 and would also make it easier to follow on which aspects these chapters focus.**

Answer:

Additional references: I included them in the discussion.

McKinney & McNamara 1991: I double-checked, they wrote the book on their own.

Fun fact: They dedicated the book to their wives “for sharing so much of their ontogenies with ours”.

**3) Bradymorphic versus tachymorphic: Schmidt (1926) introduced these terms to refer to the extreme end members of trajectories within a species which possess characteristics of earlier whorl later (in terms of heterochrony: paedomorphic) or later whorl earlier in development (in terms of heterochrony: peramorphic), respectively. Also, Beznosov and Mitta (1995) used this terminology. I feel it is worth to at least mention these terms and if these could apply to your observations.**

Answer: It is the first time I came across these two terms. Many thanks for pointing them out to me. However, these terms are used to describe intraspecific variation and not heterochronies. Since I would like to keep the Introduction very general (and heterochrony-related), I will discuss the suggested references in the discussion.

**4) Changes in the degree of intraspecific variation through ontogeny: It would speak about “life stage” or “ontogenetic stage” in this context/sentence rather than just “stage” to avoid confusion with geological stages.**

Answer: Very good point, thank you for pointing it out. I changed it.

**Line 44, Comment KDB: Bradymorphic & tachymorphic (Schmidt et al. 1926)**

Answer: see answer to email comment 3 above

**Line 59: comment KDB: I feel it would be more appropriate to give the author(s) of individual chapters in McNamara credit than just referring to them as chapter. This is to my knowledge rather a co-edited volume/book than a book written by a single author.**

Reference: McNamara & McKinney 1991 Heterochrony

Answer: see answer to email comment 2 above

**Line 210, comment KDB: Please consider discussing the long history of investigation in ammonoids on this topic (e.g., Schmidt 1926) and still a very active: (list of references)**

Answer: This comment was placed in the Methods section, which I would like to keep as short as possible. I included the suggested references in the Discussion though.

**Line 416, comment KDB: It would be appropriate to cite previous works studying intraspecific variation through ontogeny more generally.**

Text in ms: When transitions between developmental stages are accompanied by abrupt changes 416 [critical points or ‘‘Knickpunkte’’; 52, 53], developmental disparity patterns are likely to be polyphasic as well [49]

Answer: I Included some more references in the Introduction and Discussion section.

**Line 442, comment KDB: That might be true but the link between those 2 is not always straightforward - many of these classical associations are more wishful thinking than actual supported by hard evidence. Still i feel it is worth discussing. It would be worth having a look at: (list of references)**

Text in ms: There is evidence that more stable environments can generally rather be associated with slowed down growth of pedomorphic development, i.e. neoteny [2, chap. 4, 5, chap. 8].

Answer: I phrased it more carefully and added the references.
